# Supplementary material for: Magnetically mediated hole pairing in fermionic ladders of ultracold atoms
Source: Nature. 2023 Jan 18;613(7944):463–7. doi: 10.1038/s41586-022-05437-y (PMC9849138; doi:10.1038/s41586-022-05437-y)
Supplement: Supplementary file 1 — Supplementary Figs. 1–7 and references. [file 41586_2022_5437_MOESM1_ESM.pdf]

nature portfolio

https://doi.org/10.1038/s41586-022-05437-y

Supplementary information

Magnetically mediated hole pairing in fermionic ladders of ultracold atoms

In the format provided by the
authors and unedited

Supplementary Information for:
Magnetically mediated hole pairing in fermionic ladders of ultracold atoms

Sarah Hirthe[1,2,*], Thomas Chalopin[1,2], Dominik Bourgund[1,2], Petar Bojović[1,2], Annabelle Bohrdt[3,4], Eugene Demler[5], Fabian Grusdt[2,6,7], Immanuel Bloch[1,2,7], and Timon A. Hilker[1,2]

Hole correlation and binding energy

The derived formula for the binding energy (Eq. (5)) reveals the dependence of the rung hole correlator on temperature and binding energy (Fig. SS1). For lower binding energies of around $0.5J_\perp$, the $g_\mathrm{h}^{(2)}(0,1)$ correlator turns negative at the temperature of the experimental system of $T \approx 0.8J_\perp$, despite the ground state being a paired state and the correlator displaying positive values at lower temperature. For $E_b = 0.3J_\perp$, this behaviour is even more pronounced with a strongly negative signal for all but very cold temperatures. This situation happens when the binding energy is much lower than the spin gap of the system. The reason is that the spectrum features more low-lying unbound states than low-lying bound states, despite the ground state being a bound one. The low-lying states are the ones that do not break the singlet gap. A ladder with a pair and no broken singlet has all spin directions fixed. In a ladder with an unbound pair, however, there are four different combinations of spin states, that do not break the singlet gap. The unbound states are E_b higher in energy than the paired state, but for intermediate temperatures $T \gtrsim E_b$, due to the minimisation of free energy, the system will be more likely to occupy an unbound state than a paired state.

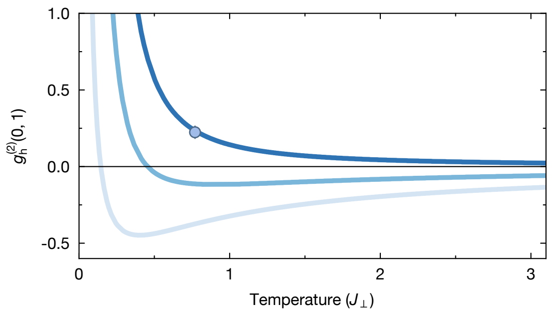

Figure S1. **Hole correlation strength $g_\mathrm{h}^{(2)}(0,1)$ as predicted by our simplified analytic model.** The rung correlation $g_\mathrm{h}^{(2)}(0,1)$ as predicted by our model (Eq. (5)) is plotted against temperature for a binding energy of $E_\mathrm{b} = 0.3J_\perp$ (light blue), $E_\mathrm{b} = 0.5J_\perp$ (blue) and $E_\mathrm{b} = 0.8J_\perp$ (dark blue). For our experimental system of $k_\mathrm{B}T = 0.77(2)J_\perp$, only the high binding energy yields a positive $g_\mathrm{h}^{(2)}(0,1)$. The marker indicates the experimental result.

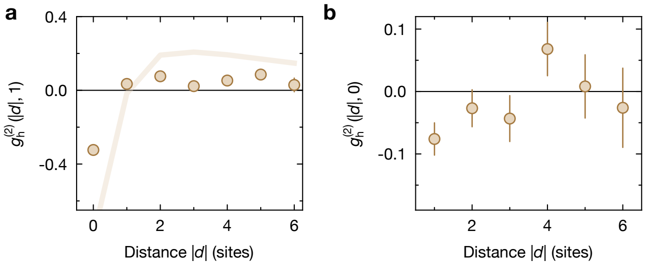

Figure S2. **Hole-hole correlation in the standard ladder. a**, Hole-hole correlation $g_\mathrm{h}^{(2)}(|d|,1)$ in the standard ladder, as shown in Fig. 2 of the main text, compared to the theoretical prediction at $k_\mathrm{B}T = 0.7\,J_\perp$ calculated using MPS (shaded line). **b**, Hole-hole correlation $g_\mathrm{h}^{(2)}(|d|,0)$ of holes in the same leg for the standard ladder with the theoretical prediction at $k_\mathrm{B}T = 0.7\,J_\perp$ calculated using MPS (shaded line).

Additional data for the standard ladder system

Figure SS2a shows the theory prediction of the hole-hole correlator for the standard ladder together with the experimental data.

Both show strong repulsion of holes on the same rung, although the experimental signal is weaker in amplitude than the numerical simulations.

The weaker repulsion that we observe in the experiment compared to theory is, most likely, attributed to inhomogeneities of the scalar potential engineered by our DMD. Factors like residual light speckles or relative position of the DMD potential with respect to the optical lattice change in time, and thus can not be detected in the time-averaged and site-resolved density. Drifts as small as a fraction of a lattice site can indeed already create a potential offset on the order of $t_\perp$, and MPS simulations show that a disorder of order $t_\perp$ on the standard ladder decreases hole repulsion by a factor 3, which is already a larger suppression than our observations. In the case of mixD ladders, however, the dynamics along the rungs are already quenched by a large potential offset $\Delta \sim U/2$ between the legs. Small fluctuations of the potential do not affect the quenched tunneling and hardly affect the spin superexchange — the corrections are in fact well within the errorbars of our system parameters — and thus have a negligible impact on our observations.

Figure SS2a is consistent with additional holes affecting the hole correlation at larger distances, similar to the mixD case discussed in the main text. In the standard case, this can be seen by the two weak maxima at distances $d = 2$ and $d = 5$. As expected, this is different to

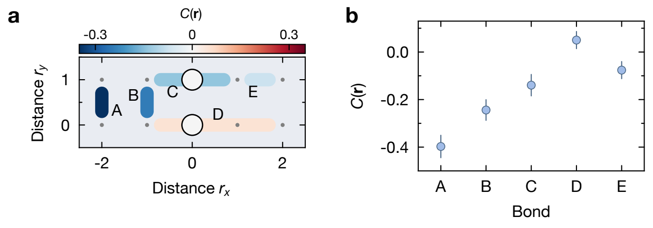

Figure S3. **Spin correlations around a hole pair.** **a**, Map of spin correlations around a hole pair in the mixed-dimensional system. The coloured bonds indicate the strength of the spin correlator, the endpoints of the bonds represent the position of the correlated sites in the frame of the hole pair, i.e. bond C denotes the spin correlation of next-nearest-neighbours across a hole pair. The data used for the analysis includes up to four holes, but no leg imbalance in the system. **b**, Quantitative values corresponding to the bonds shown in **a**. The errorbars denote one s.e.m.

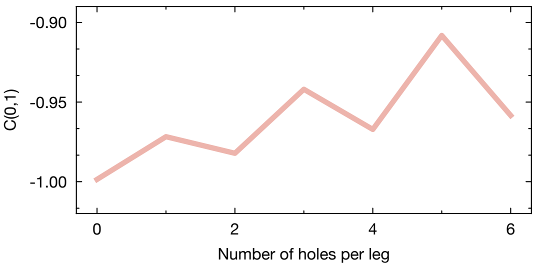

Figure S4. **Spin rung correlation vs. number of holes at low temperature.** Spin rung correlation $C(0,1)$ in the mixD ladder at a temperature of $0.1J_\perp$ depending on the number of holes in the system calculated using MPS for system length $L = 7$ and $t_\parallel = 0.6J_\perp$.

the mixD ladder, where we can explain the pattern by a first hole on the other leg being attracted, and a second hole being repelled. In the standard ladder however, already the first hole should be repelled, and a second hole gets repelled even further due to the first hole. The correlations we see are consistent with an alternating arrangement of holes on the ladder.

The repulsion of holes within the same leg can be seen in Fig. SS2b. The repulsion of holes is visible, but has a higher noise and smaller amplitude than for the mixD data. In the standard ladder the position of holes in a single leg is highly influenced by the number and position of holes in the other leg, since the repulsion of holes along the rung is much stronger than the repulsion along the leg. In the mixD system on the other hand paired holes do not influence the repulsion within a leg.

Binding energies in the standard ladder

For the standard ladder at our parameters we do not find clear signatures of binding from the DMRG calculations (see also below "*Ground state in a large system*"), i.e. our calculations are consistent with $0\,J_\perp$ binding energy. The theoretical binding energies become stronger for larger leg tunnelling $t_\parallel$, not only in the standard but also the mixD ladders [10]. For the isotropic case $t_\parallel = t_\perp$, where we find the largest binding energies in the standard Fermi-Hubbard ladders, our calculations suggest a binding energy of $0.06\,J_\perp$ for a ladder system of $L = 7$ and interactions strength of $U/t_\perp = 13.4$ similar to the experimental parameters. This corresponds to an increase in binding in the mixD ladders of about a factor of 13. Large binding energies almost up to $0.6\,J$, are predicted in the isotropic $t - J$ model, but only in parameter regimes that are not accessible from the pure Fermi-Hubbard model. In these regimes either the required ratio of t/J can not be reached based on Fermi-Hubbard ladders, or the next-nearest-neighbour tunnelling term can not be neglected, as in e.g. [34, 27, 7] (see also in Methods "*From the Fermi-Hubbard to the $t - J$ model*"). For the isotropic standard Fermi-Hubbard ladder we find, in agreement with [60], that binding energies become higher for lower interactions $U/t_\perp$ but do not exceed $0.25J$. The mixD setting we are employing circumvents these discrepancies and reaches much larger binding energies.

Spin environment around a hole pair

When a hole pair moves through the system, it locally stretches the antiferromagnetic (AFM) pattern in leg direction, analogously to spin-charge separation in 1D chains [61]. This should lead to a phase shift of π in the AFM pattern across the hole pair. Fig. SS3 shows the spin correlations $C(\boldsymbol{r}_1, \boldsymbol{r}_2)$ in the reference frame of a hole pair. The negative correlation at distance $d = 2$ over the hole pair, and the positive correlation at $d = 3$ over the hole pair are reversed in sign compared to an AFM pattern and thus consistent with such a phase jump over the hole pair. This indicates significant mobility of the hole pair along the leg.

Even vs. odd number of holes

In our finite-size system the parity of the total hole number has a significant effect on the properties of the mixD system. For odd hole number, at least one hole has to stay unpaired, because it lacks a partner. This effect can be seen in the staggered behaviour of the singlet strength at low temperature (see Fig. SS4). The leftover unpaired hole destroys singlet bonds due to its mobility along the leg direction. The bonds can be restored when an additional hole is added to the system and all holes can pair. We see a similar signature for low hole number in the experimental data (Fig. 2d).

Finite size effects

The experimental system is limited to length $L = 7$ due to the compensation of harmonic confinement and available laser power at 650 nm (see also in Methods). We thus check for possible finite size effects in the hole pairing signal. Using MPS we compute the $g_{\mathrm{h}}^{(2)}(|d|, 1)$ hole correlation for systems of up to length $L = 40$ at finite temperature (see Fig. SS5a). The main effect of small systems is a negative finite size offset for distances $|d| > 0$ while shape of the curve is mostly independent of system size. In Fig. SS5b we show the correlation stength on the same rung plotted versus system size. The reason for the vanishing finite size effects is the small size of the pairs, which are much smaller than the length of the system.

Ground state in a large systems

Since the experimental system is limited to a length of $L = 7$, we compute the ground state of a larger system of $L = 80$ containing exactly two holes using DMRG. We evaluate the bare hole correlator $\langle \hat{n}^{\mathrm{h}}_{i,\mathrm{A}} \hat{n}^{\mathrm{h}}_{j,\mathrm{B}} \rangle$ (see Fig. SS6) and compare the qualitative signatures to the experimental data. In the mixD system, we find holes predominantly on the same rung, with a small contribution on neighbouring rungs. At a distance of three rungs the correlation signal has disappeared, meaning that even in the large system holes are tightly bound. The pairs furthermore mostly occupy the central area of the system and avoid the region towards the edge, due to its kinetic energy cost. This indicates considerable degree of mobility of the hole pairs.

In the standard system holes avoid both each other and the system edge, to a level where one hole almost exclusively occupies one half of the ladder, and the other one occupies the other half. In this system size there are still no clear signs of pairing, as no attraction between the holes is visible.

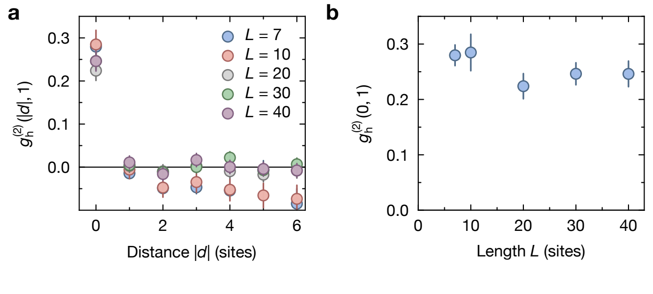

Figure S5. **Numerical hole-hole correlation in different system sizes.** **a**, Hole-hole correlation $g_{\mathrm{h}}^{(2)}(|d|, 1)$ in a mixD system of different lengths between $L = 7$ and $L = 40$ at a temperature of $k_{\mathrm{B}}T = 0.7J_{\perp}$ calculated using MPS. The hole doping is kept constant at 15 % to 30 %. The points at $|d| = 0$ are plotted against system size in **b**.

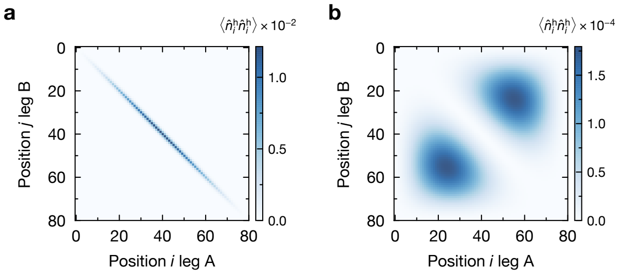

Figure S6. **Hole correlation in a larger system.** Bare hole correlation $\langle \hat{n}^{\mathrm{h}}_{i,\mathrm{A}} \hat{n}^{\mathrm{h}}_{j,\mathrm{B}} \rangle$ for a system of length $L = 80$ and $N_{\mathrm{h}} = 2$ holes. The data is calculated using DMRG with the same coupling parameters as the experimental data. **a**, MixD system. Holes are bound in tight pairs, which occupy mostly the central area of the system. **b**, Standard system. Holes avoid each other and the region towards the edge of the system.

Pair interaction: system length and coupling strength dependence

The pair-pair interaction in our MPS calculations shows the same qualitative features as in Fig. 4b when we

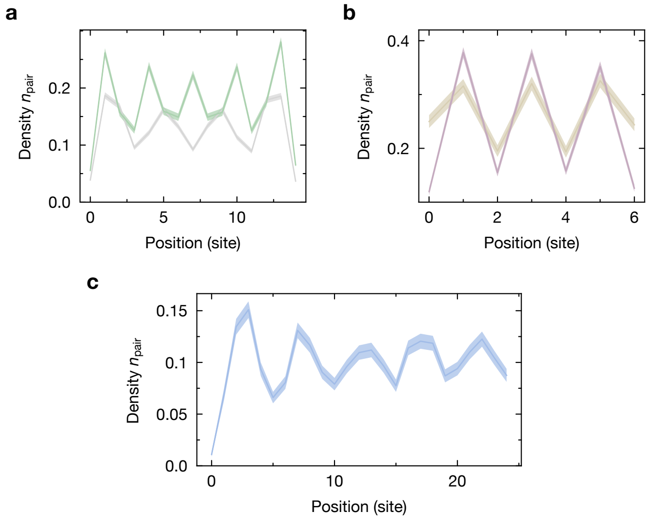

Figure S7. **Pair density for different system size and leg coupling.** **a**, Pair density at $k_{\mathrm{B}}T = 0.1J_{\perp}$ for a system length of $L = 15$ and $t_{\parallel} = 0.9J_{\perp}$ for eight holes (grey line) and 10 holes (green line) in the system. **b**, Pair density at $k_{\mathrm{B}}T = 0.1J_{\perp}$ for 6 holes in the $L = 7$ system for $t_{\parallel} = 0.6J_{\perp}$ (yellow line) and $t_{\parallel} = 1.2J_{\perp}$ (purple line). Plots **a** and **b**, are calculated using MPS. **c**, Pair density n_{pair} in the ground state for a system of length $L = 50$ and a doping of 20 % calculated using DMRG. Shaded areas denote one s.e.m.

vary the system parameters (see Fig. SS7). Increasing the system size to $L = 15$ at fixed hole number changes the wavelength of the pair density modulation, but increasing the system size at fixed hole density keeps the wavelength constant (Fig. SS7a). At hole density $n_{\mathrm{h}} = 1/3$ we find the distance between peaks in rung pair density to be 3 sites, at $n_{\mathrm{h}} \approx 1/4$ we find a distance of 4 sites, in agreement with the $L = 7$ system. Increasing the leg tunnelling (Fig. SS7b) leads to a slightly more pronounced rung pair density modulation, while the position of peaks stays the same. In particular the repulsion from the system edge increases. Note that, for simplicity, we identify the bound state as pairs of holes on the same rung, although the size of the bound state is expected to grow with increasing leg tunnelling.

Fig. SS7 shows the pair density in the ground state of a system with size $L = 50$ and 20 % doping. The density pattern is most pronounced at the edge of the system. After an initial decay the amplitude stays rather constant within the bulk of the system. We believe that this behaviour is representative for the thermodynamic limit, where we expect a charge-density-wave to form.

[61] Hilker, T. A. *et al.* Revealing hidden antiferromagnetic correlations in doped Hubbard chains via string correlators. *Science* **357**, 484–487 (2017).
